# Preliminary evaluation of an intensive integrated individual and family therapy model for self-harming adolescents

**DOI:** 10.1186/s12888-018-1947-9

**Published:** 2018-11-26

**Authors:** Moa Bråthén Wijana, Pia Enebrink, Sophie I. Liljedahl, Ata Ghaderi

**Affiliations:** 1 Department of Clinical Neuroscience, Karolinska Institutet, Stockholm, Sweden; 2 Department of Neuroscience, Child and Adolescent Psychiatry, Uppsala University, Uppsala, Sweden; 3 Department of Psychology, Lund University, Lund, Sweden

**Keywords:** Adolescents, Self-harm, Suicide, Family therapy, Intervention

## Abstract

**Background:**

To investigate the outcome of an integrated individual and family therapy (Intensive Contextual Treatment: ICT) in terms of reducing suffering and increasing functional adjustment among self-harming and/or suicidal adolescents with high symptom loads and their families.

**Methods:**

Forty-nine self-harming and/or suicidal adolescents, M_age_ = 14.6, of predominantly Swedish origin and female gender (85.7%) participated with their parents. The study had a within group design with repeated measures at pre- and post-treatment, as well as six- and twelve-months follow-ups. Self-reports were used for the main outcomes; self-harm rates, suicide attempts, parent-reported days of inpatient/institutional care, internalized and externalized symptoms, perceived stress, emotion regulation, school hours and adjustment. Secondary outcomes were levels of reported expressed emotions within family dyads, as well as parental anxiety, depression and stress.

**Results:**

From pre- to post-assessment, the adolescents reported significant reductions of self-harm (*p* = .001, *d* = 0.54) and suicide attempts (*p* < .0001, *d* = 1.38). Parent-reported days of inpatient/institutional care were reduced, as well as parent- and adolescent-reported internalizing and externalizing symptoms. Furthermore, school attendance and adjustment were improved, and the adolescents reported experiencing less criticism while parents reported less emotional over-involvement. The results were maintained at follow-ups.

**Conclusions:**

The adolescents and the parents reported improvements for the main outcomes. This treatment appears promising in keeping the families in treatment and out of hospital, suggesting that an integrative approach may be beneficial and feasible for this group.

**Trial registration:**

This study has been approved 19/12 2011, by the regional review board in Stockholm (Dnr 2011/1593–31/5).

## Introduction

Self-harm is associated with great suffering for the individual while evoking strong reactions among relatives and professionals [1–3]. Self-harm is most common during adolescence and in young adulthood, with an average age of onset between 12 and 14 years [1, 4–6]. Although self-harm has been estimated to be about three times more common in girls compared to boys [7], this ratio tends to be more equalized with increasing age [7, 8]. A systematic review of international empirical studies reported prevalence rates of 18% for non-suicidal self-injury (NSSI) and 16.1% for deliberate self-harm (DSH) among adolescents [5].

Researchers have been struggling to agree upon consistent definitions of self-harm behavior with and without suicidal intent and with determining a useful classification system [1, 4]. Numerous definitions and assessment methods complicates accurate assessments and reporting of prevalence rates. A recently established definition of NSSI is “the deliberate self-inflicted destruction of body tissue without suicidal intent for purposes not socially sanctioned” [9–12]. Examples of behaviors included are self-cutting, scratching, burning, and hitting. A definition of DSH includes any intentional self-harm, irrespective of the apparent purpose of the act. Swallowing objects, poisoning oneself, and risk-taking behavior such as jumping from heights, all fall within this definition [13].

The link between self-harm and suicide is complex and there is no established consensus whether to consider self-harm on a continuum where suicide represents one extreme [14, 15]. Self-harm, either defined as DSH or NSSI, appears to be a strong predictor of later suicide attempts and completed suicides [10, 15]. Worldwide, suicide is the second most common cause of death in young people [16]. Self-harm behaviors are also associated with a high degree of comorbidity with anxiety and depressive disorders [10, 16–18], personality disorders, and substance abuse [10].

Adolescence is a period of development characterized by physical, psychological and especially emotional changes, which also present a challenge to the family system [19–21]. Family functioning exerts a great impact on adolescents’ general well-being [22, 23]. Family conflict is considered one of the most salient predictors of suicidal events in adolescents, whereas family cohesion and adaptability are considered protective factors [24]. Individuals who engage in self-harm experience significantly lower social support, particularly from family members [25, 26]. Expressed Emotions (EE) is another well-established construct that measures the family climate. EE has been identified as a reliable psychosocial predictor of psychiatric relapse amongst vulnerable young people [27]. The main features of EE are parental criticism, blame and emotional over-involvement. High EE is typically manifested as a negative attitude directed towards the adolescent and/or intrusive behaviors in terms of over-protectiveness or self-sacrifice [28]. From the perspective of parents, having a self-harming adolescent is often associated with extreme stress, worries, guilt, shame and feelings of helplessness [3, 29]. Parental criticism, blame and emotional over-involvement (i.e. self-sacrificing or extensive controlling), might emerge or increase as a consequence of the distress caused by adolescent self-harm behavior. High EE might then lead to further increases of frequency or severity of adolescent self-harm [30].

Validation, that is, accurately expressing that a person’s experiences and emotions are listened to, understandable, and they make sense, has the effect of decreasing emotional arousal [31]. Accordingly, parents’ validation of their adolescents’ distress might be associated with more positive outcomes, such as greater satisfaction in the parent-child relationship, or improved adolescent emotion regulation [32], which in turn might reduce self-harm.

Self-harm behavior leads to tremendous individual and family costs in terms of suffering and suicide risk, and results in substantial societal costs. Given the magnitude and severity of the consequences of self-harm, effective treatments are urgently needed [33]. However, there is a lack of evidence-based treatment options for adolescents who self-harm [34–36].

When outpatient care is not sufficient to reduce symptoms and suffering, adolescents are commonly referred to inpatient care or residential treatment. The results of these treatments in Sweden rarely match their high costs [37]. Institution-based treatment leads to major interference in the adolescent life and potentially has major implication for the rest of the family, such as a rupture of the relations, a sense of failure and a perceived threat to the autonomy of the family. The prognosis for youth staying at residential homes is poor in terms of increased risk of premature death, conviction for serious crimes, and further hospitalization [37]. Until recently there has been no “gold standard” treatment for adolescents who self-harm and interventions with limited evidence have often been regarded as the best practice [38, 39]. Systematic reviews conclude that individual psychotherapy as well as medication for adolescents with self-harm and suicidal behavior shows mixed results [36, 40]. The guidelines from the National Institute of Clinical Health Excellence (NICE), do not recommend drug treatment as a specific intervention for self-harm.

The empirical data for Dialectical Behavioral Therapy (DBT), an individual- and group-based psychotherapy, related to suicidal and self-harming behavior is promising [41]. DBT was developed for suicidal women with borderline personality disorder (BPD) in the 1990’s and has been used with success more generally for people who have difficulties with emotion regulation and related difficulties [42]. Self-harm and suicidal behaviors are understood as attempts to manage uncontrollable and intensely painful emotions [42]. Self-harm among adolescents has been found to be maintained through many potent reinforcement mechanisms [1], processes which DBT aims to reduce and replace with more skillful behavioral responses [43]. Miller, Rathus and Linehan [44] have adapted DBT for use with suicidal adolescents. DBT-A [45] is sensitive to developmental and contextual considerations specific to adolescence. The emphasis is on engaging the entire family in treating self-harming and suicidal adolescents. Clinical research on DBT, in both outpatient and inpatient settings, indicates that it is an effective treatment in, for example, reducing self-harm and hospitalization [43, 46, 47]. Furthermore there is emerging data showing that DBT-A has high- acceptability ratings and retention rates. However, until very recently, studies supporting the benefits of DBT for adolescents has had methodolgical shortcomings due to significant variability regardning settings, populations and format of the treatment [48].

In a recent review, Brent and colleagues [49] advocated treatments that include family processes and support. Family therapy is another promising and effective intervention for various conditions which includes the whole family [22]. For instance, Functional Family Therapy, FFT [50] is a manualized intervention originally for families with adolescents with disruptive behavior problems [51]. FFT has also proved effective in treating adolescent depression and bipolar disorder [52, 53]. FFT is based on systemic theory, communication theory, and behavioral principles. In line with communication theory, FFT focuses on developing and strengthening responsible, supportive and positive communication strategies within the family. FFT applies behavioral principles in a manner consistent with CBT by focusing on reinforcing mechanisms. One of the core targets of FFT is to reduce EE within the family system [50]. FFT might therefore be part of an efficient treatment for adolescent self-harm.

Both DBT and FFT may be experienced as demanding treatments for self-harming adolescents and their families, and requires a certain degree of functioning of both parents and youth when offered at outpatient settings. Hawton et al. [54] suggest that for poorly motivated and dysfunctional patients, outpatient treatments should be combined with assertive outreach, such as home-based interventions, to maximize outcomes.

In summary, the implications and consequences of self-harm for the individual, family and society, lack of well-established treatments, and the early onset of the problem suggest a need for more potent and cost-effective treatments. To address this, an integrative model called Intensive Contextual Treatment for Self-Harm and Suicidality (ICT) [55], including principles from FFT, DBT-A, and Cognitive Behavior Therapy (CBT) was developed. ICT (described in more details below) is a short manual-based outpatient treatment, tailored to suit families with high symptom loads and adolescents with self-harm and suicidal thoughts and behaviors. The main aim of the present study is to investigate the outcome of ICT on self-harm behaviors and suicide attempts within a pre-post design with long-term follow-ups (six- and twelve- months’ post-treatment). The current study aimed to answer the following questions:

1. To what extent does adolescent self-harm, suicide attempts, internalizing and externalizing symptoms decrease immediately after completing the ICT treatment, and after six and 12 months?
2. Is the ICT treatment equally effective for girls and boys?
3. To what extent do the adolescents return to school or increase their attendance after completing the ICT treatment?
4. In what way do the levels EE within the family dyads, change after the ICT treatment?
5. Is the ICT treatment beneficial when it comes to the parents’ own mental health (e.g., stress, depression)?
6. To what extent does ICT prevent placements or admission to psychiatric ward?

We predicted that after the completion of ICT the adolescents would report decreased rates of self-harm and suicide attempts, and less internalizing and externalizing symptoms, reduced stress and perceived expressed emotions, increased school attendance, and improved emotion regulation abilities. We also predicted that the parents would confirm the adolescents reduced externalized and internalized symptoms. Furthermore, we predicted that the parents would report that their own mental health improved in terms of depression and anxiety symptoms as well as stress. After completing therapy, we also predicted that the parents would report reduced levels of EE within the family system.

## Method

The study has a within group design with repeated measures at pre- and post-treatment, as well as six- and twelve-months follow-ups. Ethical consideration made it impossible to adopt an experimental design including a wait-list control group, or a placebo condition.

### Participants

Adolescents aged 13–19 years were included if they had engaged in self-harm behavior on a regular basis in the past 3 months, defined as both deliberate self-poisoning and self-injury, or had expressed suicidal thoughts, threats or plans. Another inclusion criterion was that the adolescents had to live together with at least one primary caregiver. Adolescents were excluded if they reported a psychiatric disorder (e.g., schizophrenia) requiring intensive in-patient stabilization (as assessed during baseline with a semi-structured diagnostic interview), were unable to understand and speak Swedish, had severe substance abuse, or developmental disabilities. The study was conducted at Socialpsykiatriska behandlingsteamet (SPBT) a specialized team at Uppsala Child and adolescent psychiatry, Sweden from January 2012 to October 2016. A total of 60 adolescents were assessed for eligibility, of whom five did not meet the inclusion criteria, and one met the exclusion criteria. Out of 54 adolescents five were lost to follow-up due to severe eating disorder and substance use disorder, requiring other treatments (not detected during the assessment), declining further treatment or study participation.

In terms of age, we found no significant differences (*t*(52) = 0.59, *p* = .339) between the completers and the drop-out group. Similarly, we found no significant differences (*t*(42) = 0.17, *p* = .592) between completers and drop-out group regarding household monthly gross income reported by the mothers. Nor did we find any significant differences (*t*(37) = 0.01, *p* = .405) between the completers and the drop-out-group on household monthly gross income reported by the fathers. Similarly, we did not find a significant difference between completers and the dropout-up group concerning the average length of stay at psychiatric ward *(Z* = − 0.58, *N* = 52, *p* = 0.63). For the other demographic characteristics, we found no significant differences (Table 1).

**Table 1:** Demographic characteristics for the ICT, intensive contextual treatment for self-harm and dropout group. The figures are percentages, unless noted otherwise

	ICT (*n* = 49)	DROP-OUT (*n* = 5)
Adolescents’ age: *M* (*SD*)	14.6(1.3)	14.4(1.1)
Female gender	85.7	100
Adolescents’ origin^a^		
Sweden	91.8	80.0
Other countries in Europe	6.1	0
Outside Europe	2.0	20.0
Mothers’ origin^a^		
Sweden	84.1	80.0
Other countries in Europe	12.5	20.0
Outside Europe	3.4	0
Fathers’ origin^a^		
Sweden	81.6	80.0
Other countries in Europe	10.3	20.0
Outside Europe	8.0	0
Living conditions		
Mother only	34.7	20.0
Father only	2.0	0
Mother & father	46.9	80.0
Mother & father alternately	12.2	0
Other	4.0	0
Parents’ highest educational level^a^		
Elementary school	18.6	0
High school	35.4	60.0
Municipal adult education	9.3	0
University	33.7	40.0
Parents’ primary source of income^a^		
Employment	80.2	80.0
Self-employment	14.0	10.0
Parental leave	1.2	0
Unemployment or social benefits	4.7	10.0
Households’ gross income (EUR)^a^		
Reported by the mothers: M (SD)	4574(2172)	4742(3050)
Reported by the fathers: M (SD)	5284(2059)	5290(3118)
Experiencing income sufficient^a^		
Yes	60.2	60.0
No	34.1	40.0
Not reported	5.7	0
Drugs for mental health problems^a^		
Yes	77.3	30.0
No	22.7	70.0
Psychiatric diagnosis^a^		
Yes	62.1	30.0
No	37.9	70.0
Been victimized/traumatized		
Physically	8.2	0
Psychologically	14.3	20.0
Sexually	14.3	20.0
Multi traumatized	10.2	40.0
Reported yes but not described	14.3	20.0
No	38.0	0
Average days of inpatient care^a^: *M* (*SD*)	8.9(31.6)	7.5(13.3)

Note ^a^ = reported by the parents

A total of 34 (69%) of the adolescents met the criteria for a current depressive disorder, 31 (63%) met the criteria for any anxiety disorder, most commonly PTSD, and 22 (45%) met criteria for an externalizing disorder (i.e., attention deficit/hyperactivity disorder [ADHD], oppositional disorder, or conduct disorder), three met the criteria for substance use disorder and eating disorder respectively and one person met the criteria for bipolar disorder. Finally, in three cases autism spectrum disorder were suspected, but not yet confirmed. Comorbidity was common, and the most frequent combination was depression, anxiety and ADHD.

### Procedure

Participants were referred from either the child and adolescent psychiatry services (CAPS) or the social services governed by the municipality. When the eligibility criteria were met, the families were asked to provide written informed consent, before they were given instructions to complete the pre-treatment assessment. Independent clinical interviewers conducted the assessment at pre-treatment, three, six and 12 months’ post-treatment. It took an average of 1 h for the participants to fill in the questionnaires. In exceptional cases, two occasions were needed for completion to avoid fatigue, and to ensure precision in responding. Diagnostic assessment was conducted using Kiddie-Sads-Present and Life Time Version: [56]. The post assessments were scheduled to immediately follow the termination of treatment, with some variation because of naturally occurring interruptions due to holidays. The total number of therapy sessions (*M* = 35 ± 14, 77) also varied mainly depending on the distance from the clinic, which was important for accessibility. The methods for obtaining the follow-up assessment were adjusted to the capacity and motivation of the families. Most of the six-month follow-ups were conducted at the clinic or at the families’ residence with a therapist present. The families who completed the follow-up questionnaires at home and returned the assessments by mail were offered assistance by telephone and also reminders. At one-year follow up most the families answered the assessments without assistance since they by that time were familiar with the questionnaires. They were offered reminders up to three times.

### Instruments

Demographic variables such as age, daily activities, socioeconomic status, living conditions, help/support in daily life, use of medication, and exposure to traumatic event were obtained using a simple self-assessment package designed for the study. The adolescents and their families were also asked to estimate the level of school attendance, and number of days at psychiatric ward and/or residential home at all assessment points.

Self-harm was measured using the short nine-item version of the Deliberate Self-Harm Inventory (DSHI-9r), which is a modified version of Deliberate Self-Harm Inventory (DSHI) [57], adapted to adolescents by Lundh and colleagues [58] and Bjärehed and Lundh [59]. The respondents are asked if they have deliberately engaged in any of nine different forms of self-harm during the past 6 months, and are instructed to rate the number of times they have engaged in each of these behaviors on a scale from zero to six, where zero is “never” and six is defined as “more than five times”. The DSHI-9r has shown good test–retest reliability [59]. The internal consistency of the DSHI-9r in our study was good (α = .82). The time frame was changed from the past 6 months to the past month, in order to be able to detect changes during the treatment period.

The Youth Self-Report, YSR [60] is an instrument designed to measure self-reported social competence, emotional difficulties and behavioral problems during the last 6 months for adolescents aged 11–18 years. The YSR contains 112 statements scored on a three-point Likert-scale (0 = absent, 1 = occurs sometimes, 2 = occurs often). The internal consistency in a US sample was acceptable to excellent, with a Cronbach’s alpha of .76 for the DSM-oriented scales, .90 for internalizing/externalizing problems and .95 for the total scale [61]. In the present study, Cronbach’s alpha for the total scale was .70, and for internalized and externalized symptoms .73 and .65, respectively. Also, for this instrument the time frame was changed from the past 6 months to the past month.

The Child behavior checklist, CBCL [62] correspond to YSR, but intend to measure the parents’ perceptions of the adolescents’ symptoms. In this study Chronbach’s alpha was .82 (mothers) and .84 (fathers), for the internalized symptoms. For the externalized symptoms, it was .92 (mothers) and .94 (fathers).

The Perceived Stress Scale, Ten Items (PSS-10) [63] is a self-assessment that measures perceived stress during the last month on a five point Likert-scale with ten items ranging from “never” (0) to “very often” (4). Examples of questions are “In the last month, how often have you felt that you were unable to control the important things in your life?” and “In the last month, how often have you felt difficulties were piling up so high that you could not overcome them?” The PSS-10 is well established, translated into many different languages and has shown good reliability in a Swedish population, with Cronbach’s alpha of 0.84 for internal reliability [64]. In the present study, Cronbach’s alpha was 0.78 at pretreatment for the adolescents, .87 for the mothers and .83 for the fathers.

Emotion regulation was measured using Emotion Regulation Questionnaire, ERQ [65]*.* This self-assessment questionnaire is designed to capture individual differences in the habitual use of two emotion regulation strategies: cognitive reappraisal, CR and expressive suppression, ES. A higher degree of CR is considered beneficial, and seems to correlate with general well-being, life satisfaction, greater self-esteem and higher social functioning. On the other hand, a high degree of ES is considered maladaptive and is associated with more negative emotions, fewer positive relations and decreased life satisfaction. The ERQ consists of ten items on a seven-point Likert-scale ranging from “strongly disagree” (1) to “strongly agree” (7). An example of a statement about cognitive reappraisal is “When I want to feel less negative emotion (such as sadness or anger), I change what I’m thinking about.” The following statement is an example of expressive suppression; “I control my emotions by not expressing them*.*” In extensive research, ERQ has presented good psychometric properties, with adequate to good internal consistency (α = .79 for Reappraisal, .73 for Suppression) and temporal stability, 3-month test retest reliability (*r* = .69 for both scales) [65]. At pretreatment assessment alpha was .89 for the CR subscale and .60 for the ES subscale in the present study.

Questions About Family Members, QAFM [66], is a self-rating questionnaire designed to measure interactions in dyads. It consists of 30 statements of expressed emotions, on a five-point Likert-scale ranging from “almost never” to “almost always”. The QAFM is divided into four subscales; perceived criticism (e.g., “he/she is hostile towards me”.), critical comments (e.g., “I have to tell him/her to behave differently”), perceived emotional involvement (e.g., “He/she knows how I feel”), and emotional over involvement (e.g., “I can’t sleep because of him/her”). Cronbach’s alpha for the four subscales was between .61 and .89. QAFM also adequately differentiate between a clinical and non-clinical population [66]. In the present study, Cronbach’s alpha varied between .51 and .87 for the adolescents’ different subscales. Alpha for the mothers’ subscales ranged between .30 and .90 and the fathers’ between .41 and .90.

The Hospital Anxiety and Depression Survey, HADS [67] is comprised of 14 questions, and assess levels of depression and anxiety. The assessment has a four-level Likert scale and the answers are scored into two subscales, with higher points indicating higher symptom load. HADS is a widely-used instrument and has been validated for different languages and a variation of conditions. In the present study, Cronbach’s alpha for the anxiety subscale was .84 for the mothers and .82 for the fathers and alpha for the depression subscale was .76 for the mothers and .79 for the fathers.

The Family Satisfaction Survey [68] is a twelve-item questionnaire, which was completed by caregivers and adolescents to indicate how satisfied they were with treatment. The questions are answered at four-point Likert-scale at post-assessment. The instrument assesses the perception of, for example, treatment effectiveness and therapist attitude as well as the willingness to utilize the services again or to recommend it to others. Higher scores indicate greater treatment satisfaction. Cronbach’s alpha in this study was .91 for the adolescents and .79 and .75 for and the mothers and fathers respectively.

Five questions were specifically designed for this study to capture a more qualitative aspect of the school adjustment. The questions were rated on a scale from zero to four, with higher scores indicating a greater degree of adjustment. The adolescents were answering questions about how they like the school in general, how they get along with teachers, if they get good grades and evaluations, if they come on time and attend lessons and if they do their homework and hand it in on time.

### Intervention

The intervention, Intensive Contextual Treatment for Self-Harm (ICT), is a manual-based, intensive, contextual treatment. It extends over 3 months with meetings, normally at least twice a week, but more frequently when needed. The four core targets of ICT are to increase 1) the frequency of effective emotional regulation, 2) functional communication within the family, 3) school attendance or other scheduled activities, and 4) to devise a plan which clearly points to the maintenance of skills and action required in case of relapse. Throughout the treatment, a salutogenic approach is applied in the sense that much of the focus is on the functional aspects of the families and how those can be strengthened. Most meetings take place at the families’ residence. To optimize treatment and achieve synergy, both a family therapist and an individual therapist are engaged in each case providing frequent consultation to both school and social services. The model contains distinct phases with three parallel focuses; individual, family and context. Adherence is monitored via checklists [55].

The treatment was conducted by four family therapists (two males and two females) and four youth therapists (all females). The family therapists were all certified in FFT with at least Bachelor’s degree of science of social work and at least 10 years of experience from working in the social service system. Two of the family therapists also graduated as licensed psychotherapists during the study. Of the four youth therapists two were nurses specialized in psychiatry, one was a doctoral-level clinical psychologist and one was a social scientist. All the youth therapists had extensive experience of the target group, both from outpatient and inpatient care and they were also trained in DBT. To enhance ICT adherence, all therapists followed a protocol, with checklists [55]. In addition, when possible therapists also videotaped the therapy sessions for individual supervision and participated in weekly consultation team meetings and had regular meetings with a case-manager with profound knowledge of the ICT model.

#### Phase 1

The team initiates treatment with clear targets. The aims are to survey risk- and protective factors, create hope and to establish a balanced alliance towards the family members. The family therapist also formulates hypotheses regarding hierarchical patterns and relational needs. During this phase, the youth therapist uses a variation of techniques from Motivational Interviewing, MI [69]. The parents often have a higher level of motivation than the adolescent. Sometimes it requires a great deal of effort to create an alliance with the adolescent, especially if they have a history of several unsuccessful treatments. Validation of their possible resistance can be a way forward. The youth therapist often offers to attend school meetings to represent the adolescents’ interests.

#### Phase 2

The longest phase, normally lasting about 2 months. The therapeutic interventions are based on three focus areas; individual, family and context. The manual provides a framework and the content is tailored to the adolescent’s problem and the overall family situation. During phase two, the youth therapist continues the work with the adolescent and uses a wide range of techniques to accomplish behavioral changes, for example; chain analysis [43], behavioral activation [70], exposure, relationship skills and emotional regulation [43].

#### Phase 3

The third and final phase is dedicated to maintenance of acquired skills, and relapse prevention. A document is created that includes achieved goals, both individual and systemic and what specific behaviors the family members need to perform to maintain these. Also, early signs of setbacks are listed and adequate actions to prevent relapse are formulated for each involved actor. In this phase, it is important to dwell on achieved goals and skills, both behavioral and relational. In this final phase, one of the most important roles of the case manager is to keep the time frame and ensure any subsequent care can be started in a smooth transition.

### Statistical analyses

A priori power analysis indicated that a total of 50 participants would be needed to obtain a power of at least .80 for an expected medium effect size (Cohen’s *d* = 0.50) with alpha set at .05. The study was approved by the regional ethical review board in Stockholm (Dnr 2011/1593–31/5). Generalized linear mixed models (GLMM) analyses with piecewise regression were used to determine the longitudinal effect of treatment outcome. This approach has several advantages over repeated-measures ANOVA since it corrects for the correlation among repeated measurements on the same participant. Furthermore, the model handles missing data and unbalanced designs effectively using full iterative maximum likelihood estimation of model parameters. It means that no participant is excluded due to missing data, and the analysis is inherently intention to treat. Sidak post-hoc test was used to control for potential inflation of alpha due to multiple comparisons. Effect sizes (Cohens *d*) were calculated to estimate treatment efficiency. In order to examine whether the differences from pre- to post treatment and follow-up, corresponded to clinically significant improvements, participants had to report a reliable change according to Jacobson and Truax [71], and their values at post or follow-up should be within one standard deviation of the mean of the general population.

## Results

### Treatment fidelity

To ensure high treatment fidelity, therapists received regular supervision, and could discuss both the specific aspects of the cases and families, as well as delivery of the treatment with the supervisor and case manager. A checklist was devised to rate adherence to the manual, and quality of provided treatment. A maximum of five points could be given if contextual work (i.e. school meetings) as well as all the DBT- and FFT-specified interventions, related to each phase, had been delivered adequately. The mean score for fidelity for the whole study was 4.43 (*SD* = 0.58, range = 3–5), indicating high fidelity.

### Adolescents’ reports

#### Primary outcomes

Based on the adolescents’ reports there were no attempted suicides during the treatment, a finding that was confirmed by the parents and therapists. Piecewise GLMM analysis showed a statistically significant effect from pre- to post treatment with a large effect size *F*(1, 45) = 21.51, *p* < .0001, *d* = 1.38. On the other hand, from post to 6 months and 1 year follow-up we found a statistically significant deterioration and the average frequency of suicide-attempts that were nearly on the same levels as pretreatment *F*(1, 45) = 11.85, *p* = .001, *d =* 1.03.

From pre- to post treatment, the adolescents reported a 46% decrease in the rate of self-harm behavior, followed by a slight increase at the six-month follow-up and then a further reduction at the twelve-months follow-up (Table 2). The reduction from pre- to post treatment was significant with a medium effect size (Table 3). The proportions of adolescents who improved (i.e., made a reliable change), recovered (i.e., both made a reliable change and achieved a clinically significant change in terms of transferring into values within one standard deviation of the mean of the general population), remained unchanged, or deteriorated (i.e., made a reliable change in the undesired direction) based on the main outcomes at different assessment points are presented in Table 4. The general tendency as seen in Table 4 is that the proportion of adolescents classified as recovered increased from pre-treatment to all the succeeding assessment points, and the proportion of those recovered was larger for male than females. By pretreatment 71.2% of the adolescent’s report that they have been engaging in self-harming behaviors during the last month, the corresponding proportion by post treatment is 54.3% followed by a further reduction by 6 months, 50.0% and 1 year, 30.8% follow-up. Furthermore, we found significant improvement in adolescents’ self-reported internalized symptoms, level of stress, and level of emotion regulation (subscale cognitive reappraisal), with medium effect sizes (Table 4). In addition, the adolescents reported a statistically significant higher degree of school adjustment and more hours at school, at post-treatment compare to pre-treatment, with small effect sizes (Table 4).

**Table 2:** Mean and standard error of adolescents’ reported outcome variables at each time point in a GLMM model with all the tim*e points in the same GLMM model*

Variable	Pre-treatment M (SE)	Post-treatment M (SE)	6-months FU M (SE)	12-months FU M (SE)
Self-harm (DSHI-9)	15.64(2.02)	8.41(1.79)	9.04(2.19)	3.52(2.04)
YSR				
Total	79.25(3.24)	68.66(3.54)	67.10(3.82)	61.07(4.47)
Internalized symptoms	27.72(2.16)	21.20(1.86)	22.52(1.74)	19.82(1.74)
Externalized symptoms	21.14(1.36)	19.83(1.24)	17.58(1.11)	16.02(1.29)
Stress (PSS)	26.23(0.94)	22.90(1.06)	24.54(1.09)	23.84(1.30)
Emotion regulation (ERQ)				
Cognitive Reappraisal	20.10(1.23)	23.90(1.11)	22.54(0.99)	23.86(1.14)
Expressive Suppression	15.75(0.69)	14.87(0.64)	11.22(0.80)	14.55(0.96)
School				
Adjustment	11.50(0.69)	13.12(0.59)	13.14(0.71)	14.89(0.83)
Average hours at school	4.43(0.52)	5.70(0.37)	5.69(0.47)	5.73(0.53)

Note; *DSHI-9* Deliberate Self-Harm Inventory, *YSR* Youth Self Report, *PSS* Perceived Stress Scale, *ERQ* Emotion Regulation Questionnaire

**Table 3:** GLMM based effects from pre- to post assessment and during the follow-up period, respectively for the primary outcome variables reported by the adolescents

	Pre- to post-assessment	Post-assessment to 6- and 12-month follow-up
Self-Harm (DSHI-9)	*F*(1, 151) = 10.91, *p* = .001, *d* = 0.54	*F*(1, 151) = 1.52, *p* = .22, *d* = 0.20
YSR (total)	*F*(1, 152) = 14.71, *p* = .001, *d* = 0.62	*F*(1, 152) = 1.46, *p* = .23, *d* = 0.20
YSR (internalized)	*F*(1, 152) = 17.12, *p* = .001, *d* = 0.67	*F*(1, 152) = 0.68, *p* = 0.41, *d* = 0.13
YSR (externalized)	*F*(1, 152) = 1.02, *p* = .31, *d* = 0.16	*F*(1, 152) = 7.34, *p* = .01, *d* = 0.44
Stress (PSS)	*F*(1, 143) = 10.32, *p* = .002, *d* = 0.54	*F*(1, 143) = 1.69, *p* = .20, *d* = 0.22
Emotion regulation (ERQ) CR	*F*(1, 150) = 10.05, *p* = .002, *d* = 0.52	*F*(1, 150) = 1.09, *p* = .30, *d* = 0.17
Emotion regulation (ERQ) ES	*F*(1, 151) = 1.63, *p* = .20, *d* = 0.21	*F*(1, 151) = 11.65, *p* = .001, *d* = 0.56
School adjustment	*F*(1, 148) = 5.11, *p* = .03, *d* = 0.37	*F*(1, 148) = 1.15, *p* = .29, *d* = 0.18
Average hours at school	*F*(1, 117) = 4.85, *p* = .03, *d* = 0.41	*F*(1, 117) = 0.00, *p* = .98, *d* = 0.00

Note; *DSHI-9* Deliberate Self-Harm Inventory, *YSR* Youth Self Report, *PSS* Perceived Stress Scale, *ERQ* Emotion Regulation Questionnaire

**Table 4:** The percentage of participants who improved (i.e., made a reliable change), recovered (i.e., both made a reliable change and a clinically significant change in terms of transferring into values within one standard deviation of the mean of the general population), remained unchanged, or deteriorated (i.e., made a reliable change in the undesired direction) based on the main outcomes at different assessment points

	Pre- to post-assessment		Pre- treatment to 6-months FU		Pre-treatment to 1-year FU	
	Male *N* = 7	Female *N* = 42	Male *N* = 5	Female *N* = 30	Male *N* = 4	Female *N* = 22
Self-Harm (DSHI-9)						
Unchanged	71.4	64.9	40.0	56.7	50.0	50.0
Improved	0	17.5	20.0	10.0	0	4.6
Recovered	28.6	9.5	40.0	23.3	50.0	40.9
Deteriorated	0	8.1	0	10.0	0	4.5
YSR (total)						
Unchanged	57.1	92.4	60.0	83.9	33.3	69.6
Improved	28.6	2.5	20.0	9.6	66.7	4.3
Recovered	14.3	5.1	20.0	6.5	0	26.1
Deteriorated	0	0	0	0	0	0

Note; *DSHI-9* Deliberate Self-Harm Inventory, *YSR* Youth Self Report

#### Secondary outcomes

Adolescents reported a non-significant reduction of perceived criticism (PC) from their mothers, from pre-treatment (*M* = 1.81, *SE* = 0.09) to post treatment (*M* = 1.64, *SE* = 0.07), and a significant reduction *F*(1, 151) = 28.62, *p* = .001, *d* = 0.87 from post-treatment to the follow-ups (six-months: *M* = 1.20, *SE* = 0.11, twelve-months: *M* = 1.20, *SE* = 0.15). In terms of perceived emotional involvement (PEI) from their mothers, the adolescents reported a slight non-significant elevation across time.

Adolescents also reported a reduction in perceived criticism from their fathers, from pretreatment to all of the succeeding assessment points. Only the changes from post assessment (*M* = 1.54, *SE* = 0.10) to six- and twelve-months follow-up (*M* = 1.24, *SE* = 0.16, and *M* = 1.14, *SE* = 0.22, respectively) were significant *F*(1, 121) = 5.26, *p* = .02, *d* = 0.42. The changes over time for perceived emotional involvement (fathers) increased, like the mothers, but were non-significant.

### Parents’ reports

#### Primary outcomes

Both mothers and fathers reported significant changes from pre- to post-treatment on most of the outcome variables (Table 6) with moderate to large affect sizes. On the other hand, the changes from post-treatment to follow-up are generally non-significant with mainly small effect sizes (Table 6). Both mothers and fathers reported significant improvements of their adolescents’ total symptoms on CBCL, from pre- to post treatment (Table 5), with large effects for the mothers’ reports and medium for the fathers’ reports (Table 6). The same pattern of significant changes was seen on the internalized symptoms subscale of the CBCL from pre- to post treatment (Table 5) with large effect sizes for both caregivers (Table 6). Likewise, the externalized symptoms subscale of the decreased significantly based on reports from both mothers and fathers (Table 5), with medium and small effect sizes for mothers and fathers respectively. Mothers and fathers reported reduced levels of stress from pre- to post treatment (Table 5) with large effect sizes (Table 6). On HADS, mothers reported decreased levels of anxiety and depression from pre- to post treatment and further decline during the follow-up (Table 5), although the only significant change was from pre-to post, for the anxiety subscale with a medium effect size (Table 6). Fathers report on HADS revealed significantly increased levels of anxiety and depression from pre- to post treatment (Table 5), with a medium effect size (Table 6).

**Table 5:** Mean and standard error of primary outcome variables reported by mothers and fathers at each time point in a GLMM model with all the time points in the same GLMM model

	Pre-treatment	Post-treatment	6-months FU	12-months FU
	M (SE)	M (SE)	M (SE)	M (SE)
Mothers				
Child Behaviour Check List (CBCL)				
Total	57.12(3.23)	35.57(2.50)	31.22(2.83)	31.01(3.13)
Internalized symptoms	21.73(1.32)	13.61(1.06)	11.89(1.19)	11.29(1.36)
Externalized symptoms	19.43(1.41)	11.43(1.28)	9.71(1.29)	10.94(1.47)
Perceived Stress Scale (PSS)	23.98(0.86)	16.53(1.00)	16.80(0.76)	16.12(1.39)
Hospital anxiety and depression scale (HADS)				
Anxiety	11.73(0.30)	10.52(0.20)	10.38(0.29)	10.36(0.35)
Depression	8.22(0.31)	7.90(0.24)	7.71(0.28)	7.84(0.45)
Fathers				
Child Behaviour Check List (CBCL)				
Total	49.58(4.80)	33.76(3.40)	31.82(3.49)	28.68(5.71)
Internalized symptoms	19.97(1.43)	13.83(1.51)	13.43(1.48)	9.94(2.12)
Externalized symptoms	15.41(2.32)	11.11(1.53)	9.84(1.49)	9.08(2.11)
Perceived Stress Scale (PSS)	19.55(1.05)	13.47(0.93)	11.81(0.84)	14.05(1.50)
Hospital anxiety and depression scale (HADS)				
Anxiety	9.84(0.24)	10.66(0.31)	9.59(0.27)	9.78(0.37)
Depression	7.43(0.44)	7.96(0.35)	8.13(0.29)	8.27(0.43)

**Table 6:** GLMM based effects from pre- to post assessment, and during the follow-up period. Respectively for the primary outcome variables reported by the mothers and fathers

	Pre- to post-assessment	Post-assessment to 6- and 12-month follow-up
Mothers		
Child Behavior Check List (CBCL)		
Total	*F*(1, 153) = 40.23, *p* = .0001, *d* = 1.03	*F*(1, 153) = 2.97, *p* = .09, *d* = 0.28
Internalized symptoms	*F*(1, 153) = 54.90, *p* = .0001, *d* = 1.20	*F*(1, 153) = 2.50, *p* = .12, *d* = 0.26
Externalized symptoms	*F*(1, 153) = 18.17, *p* = .0001, *d* = 0.69	*F*(1, 153) = 1.32, *p* = .25, *d* = 0.19
Perceived Stress Scale (PSS)	*F*(1, 159) = 41.44, *p* = .0001, *d =* 1.02	*F*(1, 159) = 0.02, *p* = .88, *d* = 0.02
Hospital anxiety and depression scale (HADS)		
Anxiety	*F*(1, 152) = 12.54, *p* = .001, *d* = 0.57	*F*(1, 152) = 0.30, *p* = .59, *d* = 0.09
Depression	*F*(1, 153) = 0.99, *p* = .32, *d =* 0.16	*F*(1, 153) = 0.35, *p* = .56, *d* = 0.10
Fathers		
Child Behavior Check List (CBCL)		
Total	*F*(1, 115) = 15.32, *p* = .0001, *d* = 0.73	*F*(1, 115) = 0.71, *p* = .40, *d* = 0.16
Internalized symptoms	*F*(1, 115) = 23.49, *p* = .0001, *d* = 0.90	*F*(1, 115) = 0.72, *p* = .40, *d* = 0.16
Externalized symptoms	*F*(1, 112) = 5.81, *p* = .02, *d* = 0.46	*F*(1, 112) = 1.81, *p* = .18, *d* = 0.25
Perceived Stress Scale (PSS)	*F*(1, 123) = 29.30, *p* = .0001, *d* = 0.98	*F*(1, 123) = 2.92, *p* = .09, *d* = 0.31
Hospital anxiety and depression scale (HADS)		
Anxiety	*F*(1, 115) = 7.57, *p* = .01, *d* = 0.51	*F*(1, 115) = 11.43, *p* = .001, *d* = 0.63
Depression	*F*(1, 115) = 1.36, *p* = .25, *d* = 0.22	*F*(1, 115) = 0.42, *p* = .52, *d* = 0.12

#### Secondary outcomes

There was a pattern of declines on the subscales critical remarks and emotional over-involvement on the QAFM. The mothers reported a statistically significant reduction of the critical remarks towards the adolescents, from pre- (*M* = 1.78, *SE* = 0.11) to post treatment (*M* = 1.26, *SE* = 0.10), *F*(1, 150) = 17.75, *p* = .0001, *d* = 0.69. With regard to the emotional over involvement, the mothers reported significant reductions from pre- (*M* = 2.39, *SE* = 0.10) to post treatment (*M* = 1.97, *SE* = 0.10), *F*(1, 150) = 17.70, *p* = .0001, *d* = 0.69. None of the changes from post treatment to follow-up were statistically significant.

There was a slight non-significant reduction of the fathers reported critical remarks and emotional over-involvement from pre- to post treatment. Regarding the critical remarks the fathers reported significant declines from post treatment (*M* = 1.39, *SE* = 0.11) to follow-up (six-months: *M* = 1.26, *SE* = 0.11, twelve-months: *M* = 1.02, *SE* = 0.16), *F*(1, 119) = 5.06, *p* = .03, *d* = 0.41, Also the subscale emotional over involvement showed a statistically significant reduction *F*(1, 119) = 8.56, *p* = .004, *d* = 0.54, from post treatment (*M* = 2.09, *SE* = 0.10) to follow-up (six-moths: *M* = 1.93, *SE* = 0.10, twelve-months: *M* = 1.72, *SE* = 0.16).

When comparing adolescents with and without depression, anxiety, externalizing disorder and comorbidity, no statistically significant treatment effects were apparent. This might be explained by the fact that there is a heterogeneity in the sample, overlap between diagnoses and a low power.

### Patient satisfaction and service use

The adolescents’ total scores on the family satisfaction survey, FSS ranged from 2.00 to 4.00 (*M* = 3.25, *SD* = 0.50). The mothers’ scores on FSS ranged from 2.85 to 4.00 (*M* = 3.61, *SD* = 0.30), and the fathers’ scores ranged from 2.85 to 4.00 (*M* = 3.51, *SD* = 0.32).

One adolescent needed residential care during the intervention time, and another adolescent was in residential care (for 30 days) during the follow-up. The caregivers were also asked, at all assessment points to estimate how many times and days (if any) the adolescents had been in inpatient care. There was a decrease in mean number of in-patient days during the equivalent period before enrollment in the trial (*M* = 11.09, *SE* = 4.33) to post-treatment (*M* = 6.19, *SE* = 1.82) but it was not statistically significant *F*(1, 135) = 1.41, *p* = 0.24, *d* = 0.20. Finally, we found a non-significant increase of reported number of inpatient day *F*(1, 135) = 3.78, *p* = 0.06, *d* = 0.41 from post-treatment (*M* = 5.79, *SE* = 2.07) to follow-up (*M* = 11.48, *SE* = 3.97).

## Discussion

This preliminary evaluation of the ICT supports its feasibility and suggests potential treatment benefits. Adolescents reported statistically significant reductions in the frequency of self-harm and suicide attempts, internalized symptoms, and stress, and to some extent enhanced emotional regulation. Furthermore, they reported improvements in school adjustment and attendance. The parents reported statistically significant reductions in their own levels of stress. Data from parents confirmed the adolescent-reported reduction of internalized symptoms. We found mixed results regarding the parents’ own anxiety: mothers reported improvement and fathers reported deterioration. Parents reported relatively low levels of depressive symptomatology, and no statistically significant change over time. Both adolescents and parents reported high satisfaction with the treatment.

Regarding the main outcome variables (i.e., self-harm and suicide attempts) the findings in the present study are consistent with other studies in which individual and family therapy have been combined. In a trial of cognitive-behavioural family therapy by Asarnow et al. [34], the proportion of adolescents reporting self-harm at baseline was 51.1% whereas by post treatment it reduced to 31.3%. This level of reduction is comparable to our results with 71.2% at pre-treatment and 54.3% at post treatment. In the aforementioned study, suicide attempt rate was 3% at post treatment, to compare with no suicide attempts at post-treatment assessment in the present study. In a randomized controlled trial of mentalization-based treatment for self-harm in adolescents [72], the proportion of adolescents who engaged in self-harming behaviours decreased from 100 to 82% after 3 months. Aside from the fact that ICT is establishing clinical effectiveness in the current study that lacks randomization, our results can be compared to those reported in DBT-A. In a randomized controlled trial comparing DBT-A with enhanced usual care, Mehlum et.al. [73] found a reduction from an average of 1.8 episodes of self-harm/month at pre-treatment to an average of 0.8/month at post-treatment. The overall higher average rate of self-harm episodes (15.64 by pre-treatment and 8.41 by post treatment) seen in our study may be the result of using the DSHI-9r for assessment. Although frequently used in many studies, the DSHI-9r does not discriminate between severe and less severe types of self-harm. Whether the high self-harm rates seen in the participants in ICT also reflect a general higher severity is difficult to determine with reliability.

### Individual level

At the individual level, ICT seeks to promote effective emotion regulation. Emotion regulation has implications for a person’s general well-being and social relationships [65]. In the present study, the ERQ is used to determine whether the adolescents improved their emotion regulation skills after treatment. The adolescents reported statistically significant improvement on the cognitive reappraisal subscale of the ERQ from pre- to post-treatment. Specifically, they were more prone to reconstruct their thinking and re-evaluate an upcoming event in a way that potentially altered their emotional response. There was no statistically significant change from pre- to post-treatment regarding the expressive suppression subscale of the ERQ, but a reduction was found from post-treatment to follow-up, possibly indicating that they are more likely to openly express both negative and positive feelings.

The significant decline in internalized symptoms is in line with previous research findings from other similar treatment studies for adolescents with self-harming behaviours [34, 40, 43]. Although we did not find any statistically significant change on the externalized behaviour subscale of the YSR from pre- to post-treatment, such a change did occur from post-treatment to follow-up, confirmed by parent reports. Parents also reported statistically significant improvements regarding the adolescents’ externalized symptoms. The fact that several informants (adolescents, mothers, and fathers) reported the same improvements increases reliability of the results.

Stress can have a negative impact on both physical and mental health. Adolescents who engage in self-harm often report limited coping strategies and elevated levels of stress [74–76]. In the present study, the adolescents reported statistically significant reductions of perceived levels of stress from pre- to post-treatment.

In summary, the adolescents showed improvements in different areas that are likely to be related to each other.

### Family level

Distress, anxiety, exhaustion, and despair are common conditions among parents whose child engages in self-harming or suicidal behaviours. The families admitted to ICT have often been in a life-threatening and extremely distressing situation for a prolonged period of time. The clinical impression is that when parents are given the opportunity to express their fear and frustration within a professional setting, their own distress decreases. In the current study, parents reported significant improvements on most of the self-assessment measures after completing ICT. Like their adolescents, both mothers and fathers reported lower levels of perceived stress. The outcome based on data from parents had even higher effect sizes than those of the adolescents. The results based on parents’ rating of their own symptoms of depression and anxiety were not completely consistent. The mothers reported a statistically significant reduction of anxiety, whereas the fathers reported a significant increase. Mothers traditionally experience a greater responsibility when it comes to the wellbeing of the children [77]. In the context of ICT, the fathers are encouraged to share the burden to a higher degree. In other cases, the fathers had to be notified of the alarming symptoms of their adolescents, which to some extent might explain the significant elevation of anxiety.

The families seen in ICT, often describes being stuck in vicious cycles where they either directed all the frustration towards their adolescents or stopped making demands, afraid to trigger actions of self-harm. The hierarchical order is often disturbed, and the families adapt to the adolescents’ symptoms and depart from usual everyday patterns. The FFT aims at re-establishing more functional family patterns and hence reduces expressed emotion [50]. Based on t]he adolescent-reported and perceived expressed emotions on the QAFM, although declines overall were observed, the only statistically significant change is in perceived criticism from mother and fathers. Unlike the result on the other self-assessments, the improvements are predominantly seen at follow-up. Parental reports on QAFM show declines from pre- to post-treatment in all subscales. The effect seemed more pronounced in the mothers’ ratings, with fathers reporting a somewhat delayed response to treatment.

### Contextual level

Adolescents spend a great deal of time in school; hence social and academic circumstances likely influence their mental health and possibly self-harm behaviours. A prospective cohort study by Kidger and colleagues [78] showed that different aspects of school life are associated with increased risk of self-harm. The study presented examples of significant factors such as not getting along with peers and teachers, feeling disconnected from school, and poor academic achievement. In the present study, the adolescents reported statistically significant improvements regarding school adjustment and also school attendance. The ICT model has a pronounced contextual focus and it is likely that the improvements reported by the adolescents are consequences of environmental adaptation in combination with the adolescents’ improved coping skills.

Worth noting is the finding that the male participants seemed to respond better to treatment than females. One possible explanation is that males in the present study initially exhibited a higher symptom load, and consequently there is more space for improvement. There is also the possibility that the combination of DBT and FFT contribute to a better response among male participants.

When comparing the ICT treatment to similar treatment approaches, it appears superior with respect to keeping the adolescents and their families in treatment and out of hospital. ICT had a treatment completion rate of 90.7%, compared to 62% in the study by Rathus and Miller [43] where DBT-A was compared to TAU. When it comes to hospitalizations, the ICT also showed promising results. The ICT treatment targets a group of adolescents with severe psychiatric symptoms that often require intensive treatment including considerable residential care. This group is also often involved in the juvenile justice system and great societal and individual gains may be attained if these young people can avoid institutional placement. During and after the ICT treatment only two adolescents have been in residential care. It is likely that the ICT has contributed to a higher functioning among the adolescents and adaptive adjustments in the family and their broader context, which together might decrease the need for institutional placement or intensive residential care, and consequently lead to lower total costs.

This study has some limitations that must be considered. First and foremost, the non-randomized design precludes analyses of causal relationships and robust treatment effect. As mentioned in the Methods section, ethical considerations made it difficult to conduct a randomized controlled trial, specifically as the families might have been in crises and the resources in regular outpatient treatment were already exhausted. The only remining alternative in many such cases would have been full-time institutional care to ensure patient safety. Due to the risks (suicide and severe self-harm) characterizing the patients, they and their families cannot be randomized to a waitlist or a placebo condition.

There was also a considerable dropout at the follow-ups, especially at the one-year follow-up. Furthermore, even though different raters conducted the pre- and post-assessments respectively, these were not completed by blinded, completely independent assessors, which possibly could lead to biases in favour of the treatment. However, the results are mainly based on both parental and adolescent self-reports, minimizing the risk for bias. The Family Satisfaction Survey, though was collected by mail, to decrease the risk of social desirability. Another limitation was lack of objective ratings of fidelity to the treatment protocol due to some participants’ unwillingness to record the sessions. As the families were highly distressed, it was considered unethical to push harder for obtaining specific consent for recording. The limitations mentioned should also be seen in light of the strengths of the study. An important strength is the way the participants were recruited. The inclusion criteria were liberal and the ICT treatment was delivered within a defined clinical setting, strengthening the ecological validity of the study. Other strengths of the study are the prospective follow-up design, the use of standardized instruments and different informants. The response rate was also high (100%). These families were in crises and were accordingly eager to receive help. As part of the information about the study, the notion that the parents are expected to take an active role and help their youth with support from professionals was very relieving for most of the adolescents. This might have played a role in increasing the willingness of the adolescents to participate. The families were informed that declining study participation would not affect their treatment, or the resources allocated to them in any way.

### Clinical implication and further suggestions

These findings are consistent with previous research, that well-integrated and focused interventions are needed for this burdened group and that it seems crucial to include family components [25, 49]. Based on the promising findings from this preliminary evaluation of ICT, we regard the treatment program worth further examination, preferably in the context of an RCT. Evaluation of cost-benefits is a logical next step, as well as analyses of mediators and moderators. A unique contribution to the research field would be to examine the association between self-harm and expressed emotions, measured with the QAFM. Consistent with other research studies the adolescents were predominantly females (85.7%). Given the different responses to treatment between males and females, together with the fact that males have a higher risk of completing suicide, it may be important to find ways for reaching and including males in ICT. In the future, it would be valuable to compare the ICT in the present format, with an ICT version encompassing an extended transition phase, and with a control condition, to determine how to optimize and maintain long-term treatment effects. Suicide and self-harm are putting enormous strains on the economy, both locally and internationally [79–81]. Tsiachristas et al. has estimated an average hospital cost of £809 per episode of self-harm, in UK [79]. ICT represent, at least in short term a costlier alternative than regular outpatient treatment. However, regular outpatient treatment (in terms of seeing a counsellor once a week) is usually not enough for this group. ICT was initiated in collaboration between the local policymakers and clinicians as a mean to fill the gap between in- and outpatient treatment and to prevent residential care due to the extreme high cost of residential care and their unknown long-term outcome. It is difficult to estimate prevented or averted suicide attempts. The period immediately after discharge from psychiatric inpatient care has been identified as very high risk of death by suicide. There is evidence that both brief and intensive follow-ups after a suicide attempt can reduce the likelihood of a further attempt, especially if this is combined with a treatment program [79]. The present evaluation suggests that ICT might have the potential to prevent residential and inpatient care as well as suicide attempts at least in short term. A cost-benefit analysis of the present study is underway and will be presented in a separate manuscript.

## Abbreviations

ANOVA: Analysis of variance
CBCL: Child behavior checklist
CBT: Cognitive behaviour therapy
CR: Cognitive reappraisal
DBT: Dialectical behaviour therapy
DSH: Deliberate self-harm
EE: Expressed emotions
ERQ: Emotion regulation questionnaire
ES: Expressive suppression
FFT: Functional family therapy
FSS: Family satisfaction survey
GLMM: Generalized linear mixed models
HADS: Hospital anxiety and depression scale
ICT: Intensive contextual therapy for self-harm
MI: Motivational interviewing
NSSI: Non-suicidal self-injury
PSS: Perceived stress scale
QAFM: Questions about family members
RCI: Reliable change index
TAU: Treatment as usual
YSR: Youth self-report
